# Effectiveness of machine learning methods in detecting grooming: a systematic meta-analytic review

**DOI:** 10.1038/s41598-024-83003-4

**Published:** 2025-03-15

**Authors:** Marcelo Leiva-Bianchi, Nicolas Castillo, César A. Astudillo, Francisco Ahumada-Méndez

**Affiliations:** 1 Laboratory of Methodology, Behavior and Neuroscience, Faculty of Psychology, Talca, Chile; 2 Department of Computer Science, Faculty of Engineering, Talca, Chile

**Keywords:** Computational biology and bioinformatics, Psychology, Health care, Medical research

## Abstract

This study presents a systematic review (SR) and meta-analysis (MA) on the use of machine learning (ML) methods for detecting online grooming, a form of manipulation and child sexual abuse. The SR identified 33 studies from IEEE, Web of Science, Scopus, Springer, PubMed, and Google Scholar databases, and 11 ML methods were meta-analyzed for accuracy (ACC), precision (P), recall (R), and $\hbox {F}_1$ Score (F1). Multilayer Perceptron (MLP) demonstrated the highest accuracy (ACC=92%, p<0.001) and precision (P=81%, p<0.001), excelling in capturing complex, nonlinear patterns essential for analyzing nuanced online interactions. Support Vector Machine (SVM), with an ACC of 88% (p<0.001), achieved a balanced performance, characterized by high precision (P=86%, p<0.001), recall (R=74%, p<0.001), and the highest F1 score (0.79). SVM emerges as an effective algorithm, providing a robust balance across all metrics, emphasizing its adaptability and reliability in detecting nuanced grooming behaviors. This study is pioneering in meta-analyzing ML methods applied to the effectiveness in detecting grooming. The results highlight the efficacy of certain algorithms and contribute to the identification of online predators. A crucial aspect of cybersecurity for preventing child sexual abuse.

## Introduction

The grooming process, employed by those who sexually abuse children to gain their trust, is a growing issue both online and offline. Its various strategies (e.g., building trust, creating emotional bonds, psychological manipulation, gradual introduction of sexual content)^1^ have a singular objective: preparing the child for sexual abuse^2^. Its impact is significant. In 2017 alone, over 10.2 million cybertips related to the sexual exploitation of children were detected^3^. A pivotal moment in grooming research coincided with the advent of the Internet. Post-Internet, the prevailing trend focused on scrutinizing its dynamics through online datasets^4^. Two extensively utilized datasets are: (1) PAN12 (https://pan.webis.de/), and (2) Perverted Justice (PJ) (http://perverted-justice.com/). Both datasets draw from chat records where adult “decoys” pose as minors to identify potential predators. Chats in which an adult is detected engaging in grooming with a “decoy” are cataloged as cases in both datasets^5,6^. Understanding grooming and predicting it is crucial to prevent abuse, especially considering technological changes^3,7^. A recent scoping review (ScR) addresses these changes^4^. The authors categorized the literature based on the decades in which it was published (e.g., pre-Internet, post-Internet). They identified the most commonly used grooming strategies (e.g., enticements, coercion, accessibility, alcohol abuse, secrecy) and detected differences across decades. However, how can a study (e.g., ScR) fully explain a phenomenon and its process?

Systematic Reviews (SRs) are a versatile method applicable to any scientific phenomenon. For example, some articles^8,9^ claim to be Meta-Analyses (MAs). However, the one conducted by Moayedi and colleagues is not a true MA. It faces the issue highlighted by Murari^10^ regarding the use of cumulative databases. When results from different experiments or primary studies are organized within a single database, there is a risk of analyzing that database with statistical methods as if the sample comes from a single population. This increases the likelihood of error because each study has its own population and parameters. Such an approach can lead to incorrect results and conclusions. Therefore, conducting an MAs is more appropriate, as it is robust in treating each sample as part of a different population. This issue has also been identified in other studies^11^. Meanwhile, in *Scientific Reports* and in the broader scientific literature, there exists a wide range of studies on machine learning methods applied across various disciplines within multidisciplinary science. These studies encompass a variety of topics, ranging from biomedicine to astrophysics, demonstrating the potential and versatility of machine learning in addressing complex problems across multiple fields. However, a detailed review reveals a lack of research applying these methods to the study of grooming in humans, particularly in the form of meta-analyses. Only empirical studies exploring grooming behaviors in various animal species have been identified, focusing on their social functions such as care, hierarchy, or communication^12–16^.

The Support Vector Machine (SVM) is a popular algorithm for grooming detection, known for its versatility in handling diverse datasets, including categorical, quantitative, and textual attributes. SVM effectively identifies separation hyperplanes between classes, enabling accurate classification, particularly in non-linear problems with multiple dimensions.^17^. SVMs have been utilized in diverse fields including power electronics^18^, agronomy^19^, bioinformatics^20^, and software engineering^21^. Naive Bayes (NB) is a simple, fast ML model based on Bayes’ theorem, effective for low-complexity challenges, especially in text classification^22^. However, its assumption of feature independence can limit its performance in complex scenarios^23^. Logistic Regression (LogR) is a versatile algorithm for modeling the relationship between a binary dependent variable and independent variables^24,25^. It is widely used in fields like medical research, economics, and social sciences, valued for its simplicity and effectiveness in binary outcome prediction^26^. k-Nearest Neighbor (KNN) is a non-parametric classification algorithm that classifies samples based on their proximity to the k nearest neighbors in a dataset. However, its computational cost increases significantly with large datasets^27^. Random Forest (RF) is an ensemble method using multiple decision trees (DT) to improve classification accuracy^28^. While powerful, RF can be complex and requires careful selection of output functions, often demanding a large number of instances for training^23^. Adaboost (AB) enhances classification by dynamically adjusting weights on training instances, focusing on challenging samples. This method combines weak classifiers to create a robust model with high precision^29^. Gradient Boosting (GB) systematically minimizes errors using gradient descent, making it effective for complex datasets in regression and classification tasks.^30^. Multi-Layer Perceptron (MLP) is a neural network architecture that processes data through interconnected layers of neurons, optimizing parameters through backpropagation and gradient descent. MLP is effective across diverse ML tasks, from image recognition to natural language processing^31^. Convolutional Neural Networks (CNNs) excel at recognizing patterns in images and text through convolutional layers that transform data into new formats, making CNNs powerful tools for object recognition and information processing in various contexts^32^. Additional ML techniques, including Bidirectional Long Short-Term Memory (BLSTM)^33^, Gated Recurrent Units (GRU)^33^, BERT (Bidirectional Encoder Representations from Transformers)^34^, Ridge Regression (Ridge)^35^, and Recurrent Neural Networks (RNNs)^36^, are also noted in the literature.

This article contributes to the previous discussion by conducting an MA on multiple samples derived from databases used by ML methods to detect grooming. To achieve this, an SR was conducted to identify and describe ML methods employed in detecting grooming cases. Once the SR was completed, the studies amenable to MA were synthesized.

This facilitated the analysis and comparison of ML methods, addressing two key questions that structure the results in respective sections: (Q1) What are the ML techniques applied to grooming?; and (Q2) What is the best ML algorithm for detecting grooming?

It is worth noting that an MA with these characteristics has not been identified in the reviewed scientific literature thus far; it is likely to be the first of its kind. Under the title of “meta-analysis,” at least six systematic reviews have been identified regarding applications of ML in information security problems: detecting insider threats^37^, securing in small and medium-sized enterprises^38^, classifying malware^39^, enhancing vulnerability assessments and penetration testing^40^, improving anomaly detection in network intrusion systems^41^, and mitigating data security risks^42^. While their findings appear useful (e.g., SVM is identified as effective for malware classification), none of these studies conduct a statistical meta-analysis. In other words, none reports what is presented in this MA on ML: a statistical meta-analysis that includes effect sizes based on sample sizes across different populations, providing a quantitative comparison that distinguishes it from other types of reviews (e.g., qualitative, narrative, ScR, integrative).

This underscores the scientific significance of this article, building on the discussions in Ringenberg and colleagues’ ScR about how the repetition, duration, and location of grooming strategies can contribute to feature engineering (e.g., ML) applied to the identification of predators^4^. Given that the primary source of data originates from the PAN12 and PJ datasets, a fundamental assumption in our study is that MA holds relevance across diverse populations. Specifically, we consider distinct populations for each ML method applied to a randomly selected sample. This foundational assumption is critical as it forms the basis for our exploration into the generalizability of Meta-Analysis, allowing us to assess its applicability and efficacy across various datasets and ML models.

Accordingly, the objective of this study is to determine the most effective ML methods for detecting grooming through a systematic meta-analytic approach. Achieving this objective provides an explanation of current ML capabilities, guiding future research and offering practical insights to enhance child protection measures and cybersecurity interventions.

## Results: (Q1) What are the ML techniques applied to grooming?

### Studies selected for the systematic meta-analytic review

**Fig. 1:**
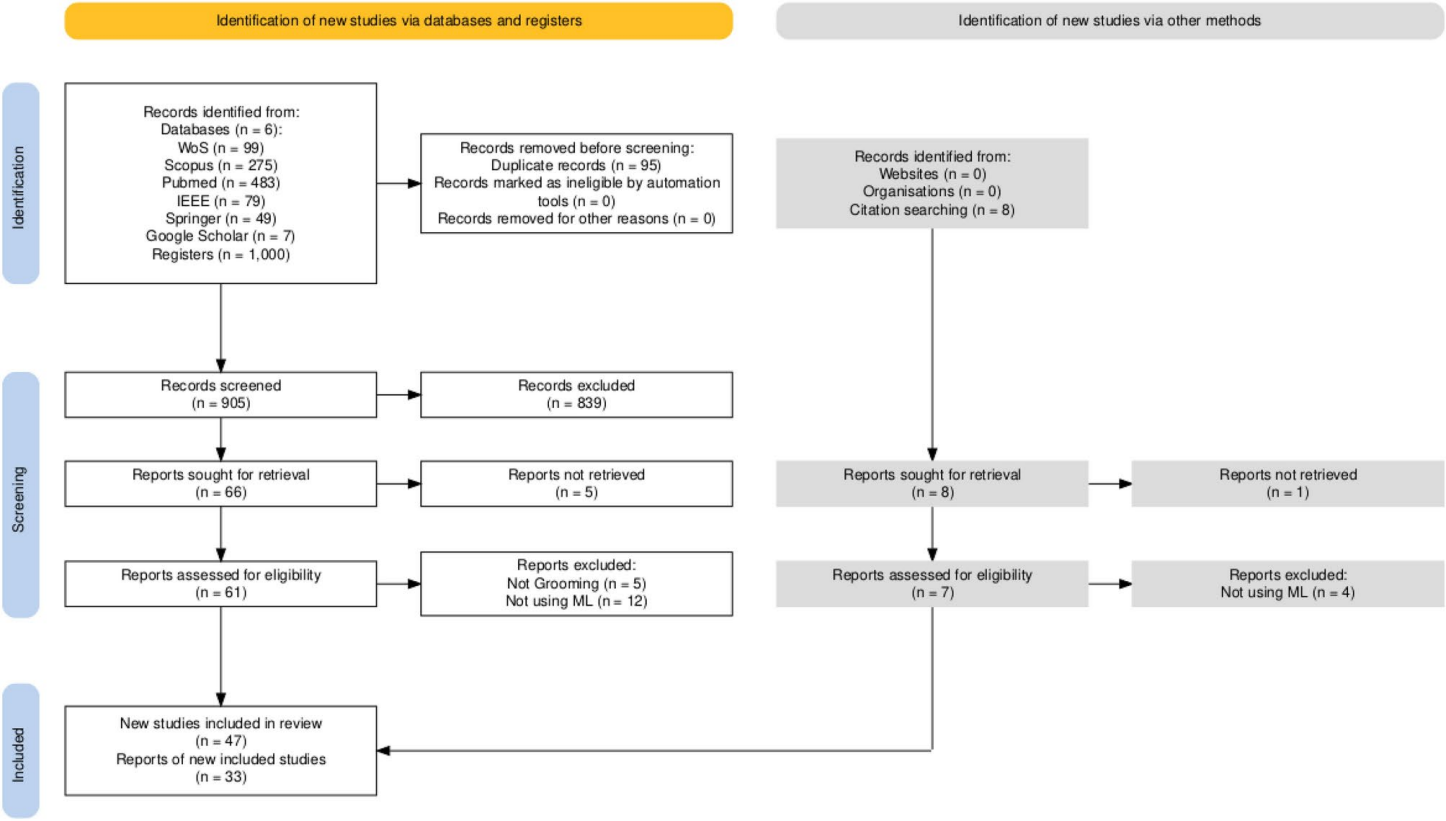
PRISMA flowchart specifying the process of selecting and including studies in the review.

The search in five electronic databases (one specialized in engineering and technology, IEEE Xplore) yielded a total of 993 articles, of which 95 duplicates were removed, and the screening process was conducted with 898 documents (title and abstract). After screening, 838 documents were excluded, and 62 moved to the eligibility phase (full analysis). Finally, 40 studies were included for the systematic review, and 26 documents for various quantitative analyses and meta-analysis. Of the 26 studies for meta-analytic analysis, 17 algorithms (e.g., SVM, NB, LogR, KNN, RF, AB, GB, MLP, and CNN), 13 datasets (e.g., PAN12, and PJ), and 8 metrics (e.g., P, R, F1, and ACC) are included. The flow diagram of the described process (PRISMA) is summarized in Figure 1.

### Description of the studies included in the meta-analytic review

Table 1 provides a comprehensive overview of various articles focused on detecting Grooming using ML techniques. It details the author and year of publication, the ML models used, the datasets employed for training and testing, and the metrics reported to assess the models’ performance. Furthermore, it provides an overview of the quality of the reviewed articles using eight questions inspired by the Critical Appraisal Skills Programme, identifying the strengths and weaknesses of the existing literature. Table 2 details the quality analysis of studies included in the systematic review. Each study is evaluated across eight quality assessment criteria (QA1 to QA8), which are detailed in the Methods section. These questions focused on essential aspects, including the clarity of the study’s research context, the details of the ML algorithm and dataset used, the rigor of the data analysis and validation methods, and the appropriateness of performance metrics for grooming detection. This assessment framework allowed us to systematically evaluate each study’s strengths and limitations, providing a clearer view of the reliability and relevance of findings across the literature^43^.

**Fig. 2:**
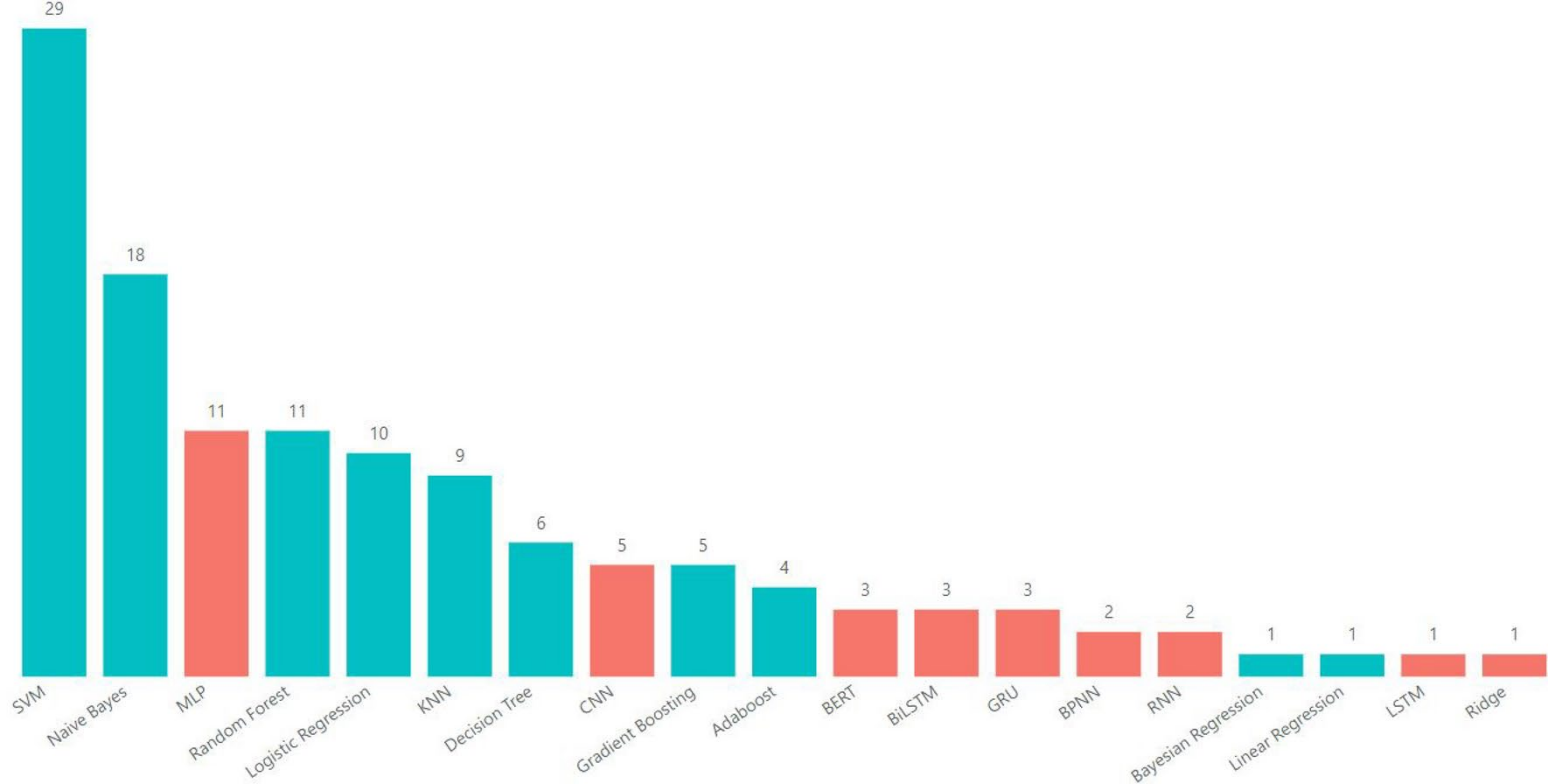
Most frequent ML algorithms for grooming detection.

**Fig. 3:**
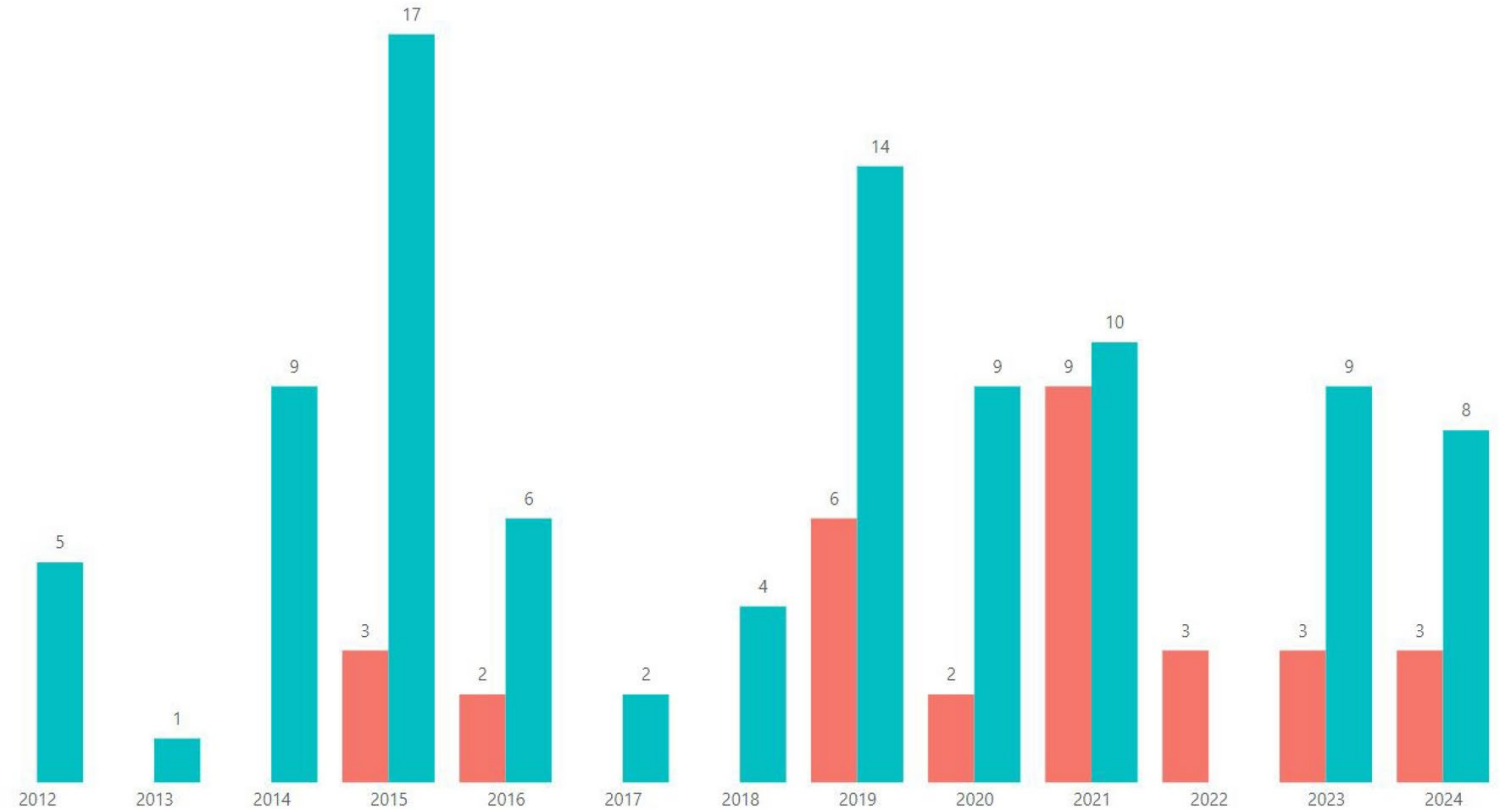
How frequent deep learning and traditional ML are utilized through the years.

Based on the grooming cases identified in the available datasets, the accuracy of the 18 algorithms used in studies reviewed from 01/01/2012 to 30/10/2024 is assessed. Among these, 10 are traditional methods (applied in 75.5% of the studies), while 8 belong to Deep Learning (DL; employed in 24.4%). Notably, since 2019, DL algorithms have gained prominence, becoming the predominant choice and reaching 100% representation in 2022 (Fig. 2, and Fig. 3). The specific methods utilized in these studies encompass a range of techniques, Dataset and Metrics.

**Table 1:** Comprehensive overview of research articles on grooming detection using ML: authors, models, datasets, and performance metrics.

Authors and Year	ML Algorithm	Dataset	Metrics	Quality
Agarwal et al. 2022^31^	MLP	PAN12	P, R, F1, F05	High
Amuchi et al. 2012^44^	NB, SVM	Private Chat	ACC	Medium
Anderson et al. 2019^45^	AB, LogR, NB, RF, SVM	PAN13, PJ	ACC	High
Andleeb et al. 2019^46^	NB, SVM	MySpace, PJ	ACC, P, F1	High
Ashcroft et al. 2015^47^	AB	PAN12, PAN13	ACC	High
Bogdanova et al. 2014^48^	SVM	CYBERSEX, NPS, PJ	ACC	Medium
Borj et al. 2019^49^	NB, RF, SVM	PAN12	ACC, P, R, F1	High
Borj et al. 2020^50^	NB, RF, SVM	PAN12	ACC, P, R, F1, F05, F2	High
Borj et al. 2021^51^	GB	PAN12	ACC, F1	High
Borj et al. 2023^52^	SVM, RF, NB	PAN12	ACC, P, R, F1, F05	High
Bours et al. 2019^35^	LogR, MLP, NB, Ridge, SVM	PAN12	P, R, F1, F05, F2	High
Cano et al. 2014^53^	SVM	PJ	P, R, F1	High
Cardei et al. 2017^54^	RF, SVM	PAN12	P, R, F05	High
Cheong et al. 2015^55^	DT, KNN, LogR, MLP, NB, RF, SVM	MovieStarPlanet	ACC, P, R, F1, F05	High
Dhouioui et al. 2016^56^	SVM	Private Chat	F1	Medium
Ebrahimi et al. 2016^57^	CNN, MLP, SVM	PAN12	P, R, F1	High
Ebrahimi, Suen et al. 2016^58^	NB, SVM	PAN12	ACC, P, R, F1	High
Faraz et al. 2024^59^	GB, KNN, SVM	PAN12	ACC, P, R, F1, F2, F05	High
Fauzi et al. 2020^60^	DT, KNN, LogR, MLP, NB, RF, SVM	PAN12	ACC, P, R, F1, F05	High
Fauzi et al. 2023^61^	SVM	PAN12	ACC, P, R, F1, F05	High
Gunawan et al. 2016^62^	KNN, SVM	LITEROKA, PJ	ACC	Medium
Hamzah et al. 2021^33^	BiLSTM, GB, GRU	Chat not specified, PAN12	ACC	Medium
Isaza et al. 2022^63^	CNN	PAN12	ACC, P, R, F1, S	Medium
Kim et al. 2020^36^	RNN	PAN12	P, R, F1, F05	Medium
Kirupalini et al. 2021^64^	AB, KNN, NB, RF, SVM	FUGLY, IRC, PJ	ACC, P, R, F1	High
Melleby 2023^65^	BERT	PAN12, AIBA AS	P, R, F1	High
Michalopoulos et al. 2014^66^	KNN, NB, SVM	PJ	ACC	Medium
Milon et al. 2022^67^	MLP	IRC, PJ	F1	High
Misra et al. 2019^68^	CNN	PAN12, PJ	F1	Medium
Mohammed et al. 2024^69^	SVM, NB, RF	PAN12	ACC, P, R, F1	Medium
Morris et al. 2013^17^	SVM	PAN12	P, R, F1	High
Munoz et al. 2021^70^	CNN	Chat not specified, PAN12	ACC, R, F1	High
Ngejane et al. 2021^71^	BLSTM, GB, LogR, MLP	PAN12	ACC, P, R, F1	High
Nyrem et al. 2023^72^	NB, DT, KNN, LogR, MLP, RF	PAN12	ACC, P, R, F1	High
Pandey et al. 2012^73^	SVM	Omegle, PJ	ACC	Medium
Parapar et al. 2014^74^	SVM	PAN12	P, R, F1	High
Peersman et al. 2012^75^	SVM	PAN12	P, R, F1	High
Pranoto et al. 2015^25^	LogR	PJ	ACC, P, R	Medium
Preub et al. 2021^76^	MLP	PAN12	P, R, F05, F2	High
Ringenberg et al. 2019^2^	CNN	PJ	NA	Medium
Suhartono et al. 2019^77^	BPNN	PJ	ACC	High
Vartapetiance et al. 2014^78^	NB, SVM, DT	PAN12	P, R, F1	High
Vogt et al. 2021^34^	BERT	CHATCODER, PAN12	P, R, F1	High
Waezi et al. 2024^79^	SVN, RNN, LSTM, GRU	PAN12	F2, F05	High
Zambrano et al. 2019^80^	LR	PJ	ACC	Medium
Zuo et al. 2018^81^	AB, LogR, NB, RF	Chat not specified, PAN13	ACC	Medium
Zuo et al. 2019^82^	BPNN	Chat not specified, PAN13	ACC	Medium

**Table 2:** Results of the quality analysis performed for each of the studies in the SLR.

Study	QA1	QA2	QA3	QA4	QA5	QA6	QA7	QA8	Total
Agarwal et al. 2022^31^	1	1	1	1	1	1	1	0	7
Amuchi et al. 2012^44^	1	1	1	1	0	0	1	0	5
Anderson et al. 2019^45^	1	1	1	1	1	0	1	1	7
Andleeb et al. 2019^46^	1	1	1	1	1	1	1	1	8
Ashcroft et al.^47^	1	1	1	1	1	0	1	1	7
Bogdanova et al. 2014^48^	1	1	1	1	0	0	1	1	6
Borj et al. 2019^49^	1	1	1	1	0	1	1	1	7
Borj et al. 2020^50^	1	1	1	1	1	1	1	0	7
Borj et al. 2021^51^	1	1	1	1	1	1	1	0	7
Borj et al. 2023^52^	1	1	1	1	1	1	1	1	8
Bours e al. 2019^35^	1	1	1	1	1	1	1	1	8
Cano et al. 2014^53^	1	1	1	1	1	1	1	1	8
Cardei et al. 2017^54^	1	1	1	1	1	1	1	0	7
Cheong et al. 2015^55^	1	1	1	1	1	1	1	0	7
Dhouioui et al. 2016^56^	1	1	0	0	1	0	1	0	4
Ebrahimi et al. 2016^57^	1	1	1	1	1	1	1	0	7
Ebrahimi, Suen et al. 2016^58^	1	1	1	1	1	1	1	0	7
Faraz et al. 2024^59^	1	1	1	1	1	1	1	1	8
Fauzi et al. 2020^60^	1	1	1	1	1	1	1	0	7
Fauzi et al. 2023^61^	1	1	1	1	1	1	1	1	8
Gunawan et al. 2016^62^	1	1	1	1	1	0	1	0	6
Hamzah et al. 2021^33^	1	1	1	0	1	0	1	0	5
Isaza et al. 2022^63^	1	1	0	1	1	1	1	0	6
Kim et al. 2020^36^	1	0	1	0	1	1	1	0	5
Kirupalini et al. 2021^64^	1	1	1	1	1	1	1	1	8
Melleby 2023^65^	1	1	1	1	1	1	1	1	8
Michalopoulos et al. 2014^66^	1	1	0	0	0	0	1	1	4
Milon et al. 2022^67^	1	1	1	1	1	1	1	0	7
Misra et al. 2019^68^	1	1	1	0	1	1	1	0	6
Mohammed et al. 2024^69^	1	1	1	1	1	1	1	0	7
Morris et al. 2013^17^	1	1	1	1	1	1	1	1	8
Munoz et al. 2021^70^	1	0	1	1	1	1	1	1	7
Ngejane et al. 2021^71^	1	1	1	1	1	1	1	1	8
Nyrem et al. 2023^72^	1	1	1	1	1	1	1	1	8
Pandey et al. 2012^73^	1	1	0	1	1	0	1	0	5
Parapar et al. 2014^74^	1	1	1	1	1	1	1	0	7
Peersman et al. 2012^75^	1	1	1	1	1	1	1	0	7
Pranoto^25^	1	1	1	1	1	0	1	0	6
Preub et al. 2021^76^	1	1	1	1	1	1	1	1	8
Ringenberg et al. 2019^2^	1	1	1	1	0	0	1	0	5
Suhartono et al. 2019^77^	1	1	1	1	1	0	1	1	7
Vartapetiance et al. 2014^78^	1	1	1	1	1	1	1	0	7
Vogt et al. 2021^34^	1	1	1	1	1	1	1	1	8
Waezi et al. 2024^79^	1	1	1	1	1	1	1	1	8
Zambrano et al. 2019^80^	1	0	1	1	1	0	1	0	5
Zuo et al. 2018^81^	1	1	1	1	1	0	1	0	6
Zuo et al. 2018^82^	1	1	1	0	1	0	1	0	5

### Indicators of effectivity

In evaluating the capacity of a ML method to accurately classify grooming cases, we identified five crucial indicators: Accuracy (ACC), F-1 score (F1), Precision (P), Recall (R), and Specificity (S). This study employs a comprehensive meta-analysis approach, systematically examining each of these indicators. Through this meta-analysis, we aim to provide a nuanced and thorough assessment of the model’s classification performance, shedding light on its effectiveness in addressing the challenges posed by grooming cases. This approach ensures a robust and holistic understanding of the method’s capabilities, facilitating informed decisions regarding its applicability and reliability in real-world scenarios. While these performance metrics, such as accuracy and F-score, are standard in Machine Learning research, we have included them for the benefit of readers from related fields, such as cyberbullying research, who may not be as familiar with these concepts. This ensures clarity and facilitates a better understanding of how the results presented in this study were derived, enhancing the accessibility and reproducibility of our work.

**Fig. 4:**
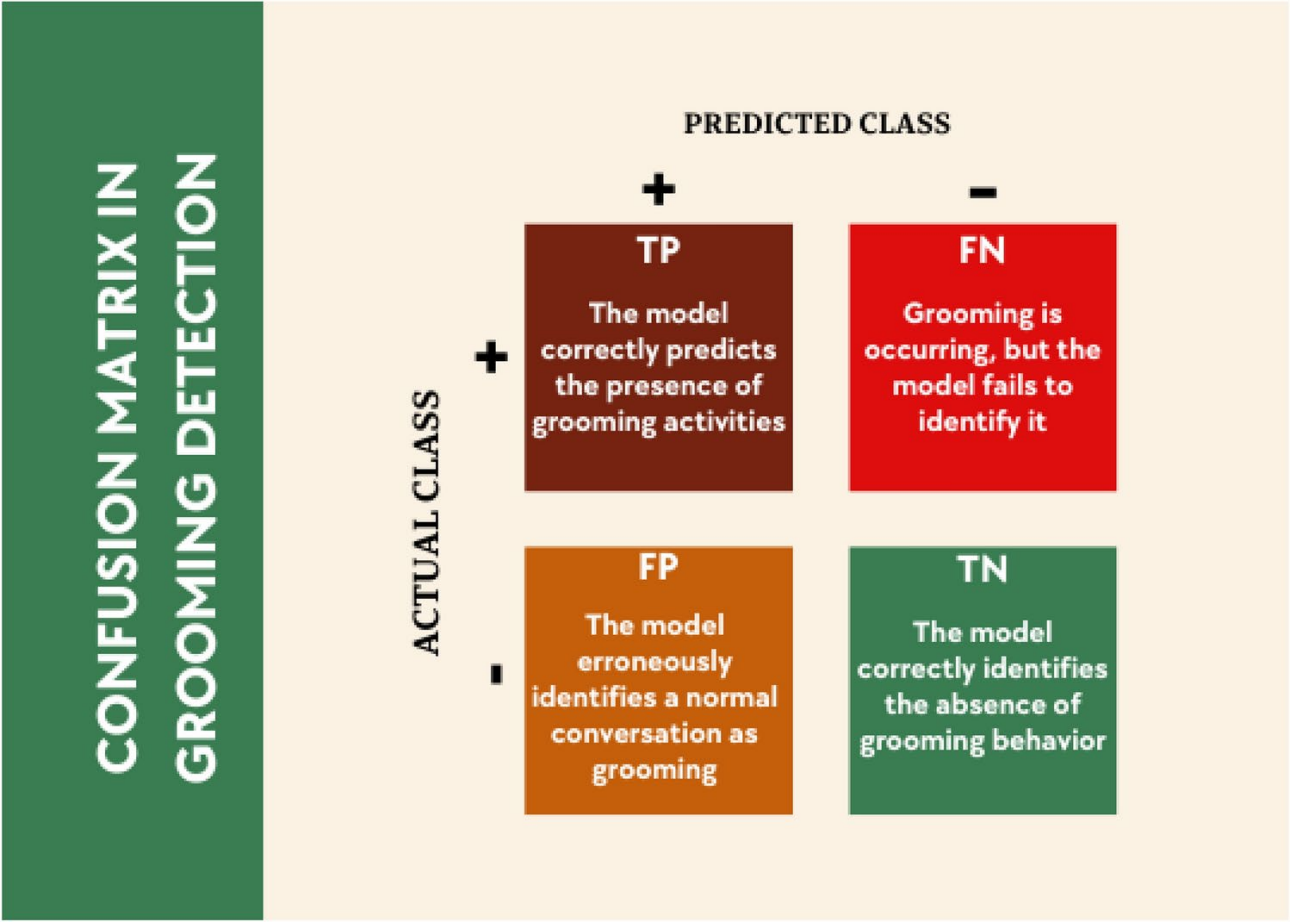
Confusion matrix in the context of grooming detection.

The above-mentioned metrics derive from the so-called confusion matrix, which details the performance of classification algorithms. Figure 4 illustrates the structure of the confusion matrix and how the most common metrics are derived. Rows denote the actual labels of the dataset, while columns identify labels estimated by the classifier. Each cell outlines the instances with a combination of real labels and predictions made by the classifier. Each cell describes the instances according to the combination of actual labels and the model’s predictions. The main diagonal elements correspond to correctly classified instances (True Positives and True Negatives), whereas the off-diagonal cells document errors made by the system (False Positives and False Negatives). This structure allows for the calculation of key metrics such as Precision, Recall, and Specificity, providing a comprehensive understanding of the model’s capacity to identify and distinguish grooming cases.

True positives (TP) are instances correctly predicted as belonging to the positive class, while false positives (FP) are cases incorrectly classified as positive when they actually belong to the negative class. True negatives (TN) represent instances correctly identified as negative, and false negatives (FN) are positive cases mistakenly labeled as negative. This breakdown provides a clear understanding of the classifier’s performance in distinguishing between positive and negative classes. In grooming detection, the positive class refers to correctly identified grooming activities, while the negative class involves the accurate identification of non-grooming behavior. The distinction between errors in predicting positive and negative classes is crucial. FN represents a failure to detect actual grooming, posing a significant risk, as it allows perpetrators to go unnoticed. Conversely, FP involves mistakenly identifying normal conversations as grooming, leading to potential misunderstandings but generally considered less severe than FN. Managing these distinctions is essential for optimizing grooming detection systems.

One of the fundamental and widely utilized performance metrics for assessing a classifier is accuracy (ACC). As denoted by Eq. (1), this metric was applied in 77 instances. It is computed by dividing the sum of the diagonal elements of the confusion matrix by the total number of instances examined. This straightforward yet pivotal measure offers a clear indication of the model’s overall correctness in its predictions.

1 $$\begin{aligned} ACC = \frac{TP + TN}{TP + FP + FN + TN} \end{aligned}$$

Having been reported 67 times as a benchmark for effectiveness, Precision (P) evaluates quality by considering the number of accurately detected positive instances. This indicator, detailed in Eq. (2), provides insights into the precision of a classification model through the identification of positive cases within the subset labeled as positive.

2 $$\begin{aligned} Precision = \frac{TP}{TP + FP} \end{aligned}$$

Recall (R), reported 66 times in the articles investigated, represents the percentage of positive instances detected compared to the total positive instances in the sample. Calculated using Eq. (3), this indicator derives from the ratio of TPs to the sum of TPs and FNs, quantifying the model’s capacity to accurately capture all positive instances.

3 $$\begin{aligned} Recall = \frac{TP}{TP + FN} \end{aligned}$$

The $F_1$ Score (F1) is the most frequently cited indicator in selected papers on grooming detection with 115 occurrences.

4 $$\begin{aligned} F_1 = \frac{2 \cdot P \cdot R}{P + R} \end{aligned}$$

The F1 Score, formally defined by Eq. (4) considers the harmonic mean of P and R, giving equal weight to both factors. The F1 Score is particularly useful when there is a need to balance precision and recall without favoring one over the other.

Finally, Specificity (S) indicates the probability of detecting negative instances among the total negative instances in the sample and was reported only once in the context of grooming detection (see Eq. (5)); therefore, it was not considered in the meta-analysis.

5 $$\begin{aligned} Specificity = \frac{TN}{TN + FP} \end{aligned}$$

## Results: (Q2) What is the best ML algorithm for detecting grooming?

### SR and MA of ML methods for grooming detection

The first scenario assumes that each ML method would perform similarly in detecting grooming, regardless of the type or database used for training. Differences in algorithm effectiveness between studies would be attributed solely to variability in the data samples, not differences in efficacy. Therefore, the studies vary similarly, meaning they are more homogeneous, and their effect on predicting accuracy is consistent.

A second scenario considers that the performance of the ML method varies between studies not only due to random selection of observations but also due to other factors (e.g., algorithm type, database size, data quality, study design). As a result, they vary differently, increasing the heterogeneity in the meta-analysis, making the effect random.

#### Meta-analysis for accuracy

Table 3 presents a meta-analysis focused on the performance of 10 algorithms based on ACC. The algorithms analyzed include BiLSTM, GB, MLP, DT, SVM, LogR, NB, RF, KNN and AB. It is crucial to consider that accuracy is focused on correctly classified elements, a task that may be challenging when faced with imbalanced classes.

The accuracy scores show that BiLSTM leads with an ACC of 0.95, closely followed by GB and MLP. This high level of accuracy suggests that BiLSTM, GB, and MLP are particularly effective for detecting grooming. The variability, represented by confidence intervals, is relatively narrow for these top-performing algorithms, indicating a consistent performance across different studies. It is crucial to consider that accuracy is focused on correctly classified elements, a task that may be challenging when faced with imbalanced classes.

The $\text {I}^{2}$ statistic, which measures the percentage of total variation across studies due to heterogeneity rather than chance, is extremely high (above 95%) for all algorithms. This suggests considerable variability in the results across different studies, which could be due to factors like varying data sets, experimental conditions, or implementation specifics.

All algorithms show p-values less than 0.001, indicating that the reported accuracies are statistically significant. However, the high $\text {I}^{2}$ values call for cautious interpretation, as the context of each study may strongly influence the results.

The Fail-Safe N values are notably high for algorithms like SVM, MLP, NB, and LogR, indicating that a large number of unpublished or undiscovered studies with null results would be needed to invalidate these findings. This suggests robustness in the reported accuracies for these algorithms.

In terms of the number of articles and studies, SVM and NB are the most researched, with 10 articles each. This extensive research base might contribute to their higher accuracy and reliability.

AB, with the lowest ACC of 0.62 and a relatively low $\text {I}^{2}$ statistic, stands out as having less variability across studies but significantly lower performance compared to the other algorithms.

#### Meta-analysis for precision

Seven meta-analyses were conducted to assess the P indicator (see Table 8). The RF algorithm emerged as the most efficient, achieving 99% accuracy. These statistically significant results affirm their reliability.

SVM also demonstrates high precision (0.86), with a variability range of [0.80, 0.93]. Like RF, it has a 100% $I^2$ statistic and a significant p-value, along with a very high Fail-Safe N. This suggests that SVM is reliably precise across different studies, despite the high heterogeneity.

Furthermore, MLP has a precision of 0.81 with a wider variability range [0.71, 0.92] and a nearly 100% $I^2$ statistic. The p-value is less than 0.001, and the Fail-Safe N is large, indicating significant results and robustness against bias.

Logistic Regression (LogR), Decision Tree (DT), Naive Bayes (NB), and K-Nearest Neighbors (KNN) show lower precision values (0.76, 0.75, 0.73, and 0.68, respectively). KNN, in particular, shows the widest variability range [0.35, 1.00], indicating the most inconsistency in precision across studies.

The meta-analysis presented in Table 8 reveals that, when focusing on the precision metric, RF and SVM are the most precise algorithms among those analyzed, with RF slightly outperforming SVM. The high $I^2$ values across all algorithms suggest substantial variability in precision across different studies, emphasizing the context-dependent nature of these algorithms’ performances. Despite this variability, the significant p-values and high Fail-Safe N values for each algorithm indicate that the results are statistically robust and not overly influenced by publication bias. In the context of grooming detection, where precision is crucial to minimize FPs, these findings are particularly relevant.

#### Meta-analysis for recall

Eight meta-analyses were conducted to evaluate the R indicator. The ML method showing the best performance was LogR (80% sensitivity), followed by MLP (75%) and SVM (74%). In contrast, RF showed low performance, evidenced by a p-value greater than 0.05, suggesting that the results could be due to chance rather than the algorithm’s effectiveness. High heterogeneity and variability were also found in the results. Similar to previous indicators, no publication bias was detected using Fail-Safe N (see Table 5)

#### Meta-analysis for F1

The meta-analysis presented in Table 6 focuses on comparing eight algorithms based on their F1 Scores, which is a measure of a test’s accuracy. The algorithms included are MLP, SVM, LogR, NB, DT, CNN, KNN, and RF.

The F1 Scores range from 0.30 for RF to 0.79 for both MLP and SVM, indicating a wide disparity in performance across these algorithms. Both MLP and SVM show high effectiveness with similar F1 Scores, but SVM is supported by a larger number of articles and studies, potentially indicating more robust evidence. The variability column, which represents confidence intervals, shows a range for each F1 Score. Algorithms like MLP, SVM, and LogR exhibit relatively tight confidence intervals, suggesting more consistent performance across different studies. In contrast, RF has a wide interval and even includes a negative lower bound, highlighting uncertainty in its performance.

The $I^2$ statistic, a measure of heterogeneity, is very high (near 100%) for all algorithms, indicating substantial variability between studies. This suggests that the F1 Scores reported for these algorithms may be influenced by factors specific to individual studies, such as dataset characteristics or experimental setups.

The p-values are less than 0.001 for all algorithms except RF, which has a p-value of 0.110. This indicates that the results are statistically significant for all but RF, where the significance is questionable.

The Fail-Safe N values, which indicate the number of additional studies with null results needed to bring the overall effect size to an insignificant level, are notably high for SVM, MLP, and NB. This suggests that the results for these algorithms are robust against the potential publication bias.

Lastly, the number of articles and studies varies across algorithms, with SVM, NB, and MLP being the most studied, which could have contributed to their higher F1 Scores due to more extensive testing and optimization in various scenarios.

**Table 3:** Algorithm performance rankings based on the Accuracy (ACC) in the meta-analysis, arranged from the highest to the lowest.

Algorithm	**ACC**	Variability	${\textbf {I}}^{{\textbf {2}}}$	p	Fail-Safe N	# Articles	Studies
BiLSTM	0.95	[0.90, 0.99]	99.57%	<0.001	753082	2	3
GB	0.94	[0.88, 0.99]	99.94%	<0.001	3153623	3	4
MLP	0.92	[0.86, 0.99]	99.97%	<0.001	8228112	3	5
DT	0.89	[0.83, 0.95]	99.83%	<0.001	835064	2	4
SVM	0.88	[0.81, 0.95]	100.00%	<0.001	78775652	10	12
LogR	0.83	[0.73, 0.93]	99.98%	<0.001	9713684	6	8
NB	0.83	[0.74, 0.92]	99.99%	<0.001	55607446	10	12
RF	0.81	[0.67, 0.96]	99.99%	<0.001	4470728	5	5
KNN	0.81	[0.59, 1.03]	99.98%	<0.001	983283	4	6
AB	0.62	[0.57, 0.68]	95.00%	<0.001	5397	3	3

**Table 4:** Meta-analysis for the precision.

Algorithm	Precision	Variability	${\textbf {I}}^{{\textbf {2}}}$	p	Fail-Safe N	# Articles	Studies
RF	0.99	[0.97, 1.01]	100.00%	<0.001	2023671472	4	4
SVM	0.86	[0.80, 0.93]	100.00%	<0.001	717650281	13	15
MLP	0.81	[0.71, 0.92]	99.99%	<0.001	9954497	7	9
LogR	0.76	[0.58, 0.95]	100.00%	<0.001	314960983	5	7
DT	0.75	[0.66, 0.84]	99.93%	<0.001	1192338	3	5
NB	0.73	[0.56, 0.90]	100.00%	<0.001	12707409	8	10
KNN	0.68	[0.35, 1.00]	99.99%	<0.001	3315530	2	4

**Table 5:** Meta-analysis for the recall.

Algorithm	Recall	Variability	${\textbf {I}}^{{\textbf {2}}}$	p	Fail-Safe N	# Articles	Studies
LogR	0.80	[0.68, 0.92]	99.95%	<0.001	932729	5	7
MLP	0.78	[0.64, 0.92]	99.99%	<0.001	10882300	7	9
SVM	0.74	[0.64, 0.83]	99.99%	<0.001	17141834	12	14
CNN	0.73	[0.68, 0.77]	99.99%	<0.001	8870121	3	3
NB	0.70	[0.52, 0.89]	100.00%	<0.001	7887378	7	9
KNN	0.64	[0.25, 1.02]	100.00%	<0.001	556887	2	4
DT	0.56	[0.40, 0.72]	99.97%	<0.001	268680	3	5
RF	0.25	[-0.11, 0.60]	100.00%	0.172	91681	4	4

**Table 6:** Meta-analysis for the F1 Score.

Algorithm	F1 Score	Variability	${\textbf {I}}^{{\textbf {2}}}$	p	Fail-Safe N	# Articles	Studies
MLP	0.79	[0.67, 0.90]	99.99%	<0.001	6853926	7	9
SVM	0.79	[0.72, 0.87]	99.99%	<0.001	24348121	13	15
LogR	0.76	[0.58, 0.86]	99.97%	<0.001	1437624	5	7
NB	0.65	[0.46, 0.84]	100.00%	<0.001	4230599	8	10
DT	0.62	[0.51, 0.74]	99.95%	<0.001	390653	3	5
CNN	0.54	[0.28, 0.80]	100.00%	<0.001	2484955	3	3
KNN	0.45	[0.32, 0.57]	99.98%	<0.001	60794	2	4
RF	0.30	[-0.07, 0.67]	100.00%	0.110	259873	4	4

**Fig. 5:**
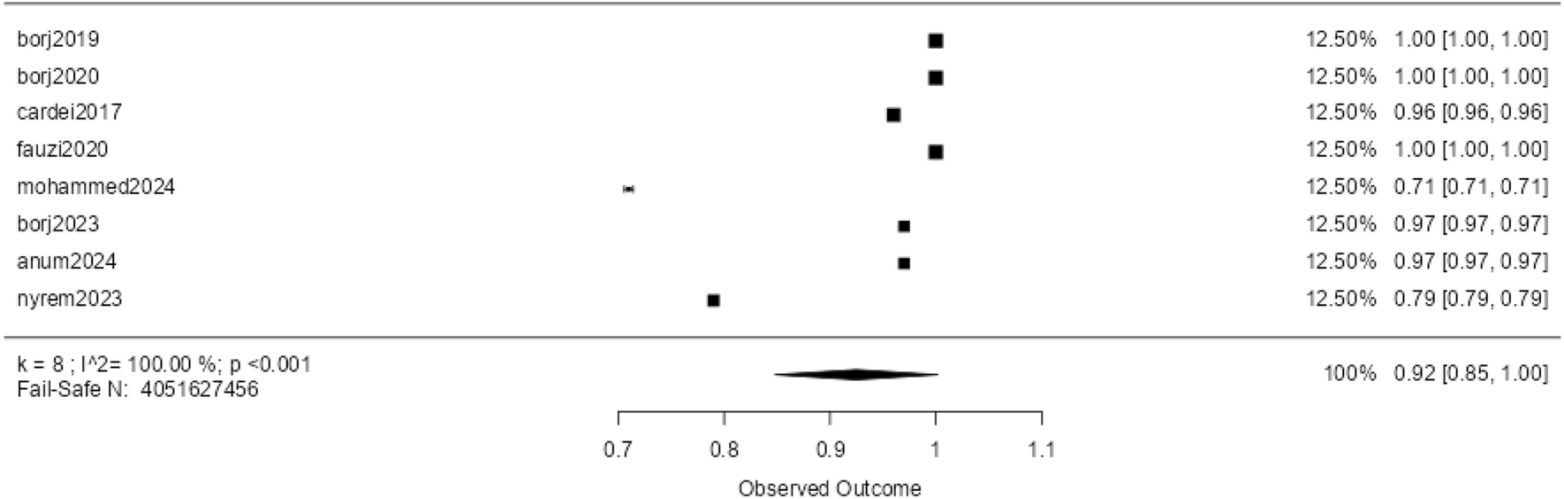
Forest plot for RF using metric P.

Figure 5 presents a forest plot for the Random Forest (RF) algorithm using the precision metric (P), showing high effectiveness across four studies. Three studies report 100% precision, while one study^54^ shows 96% precision. The solid black squares in the plot indicate minimal error, resulting in a meta-analytic estimate of 0.99 for RF. However, the small sample size suggests the need for further research to confirm RF’s precision.

**Fig. 6:**
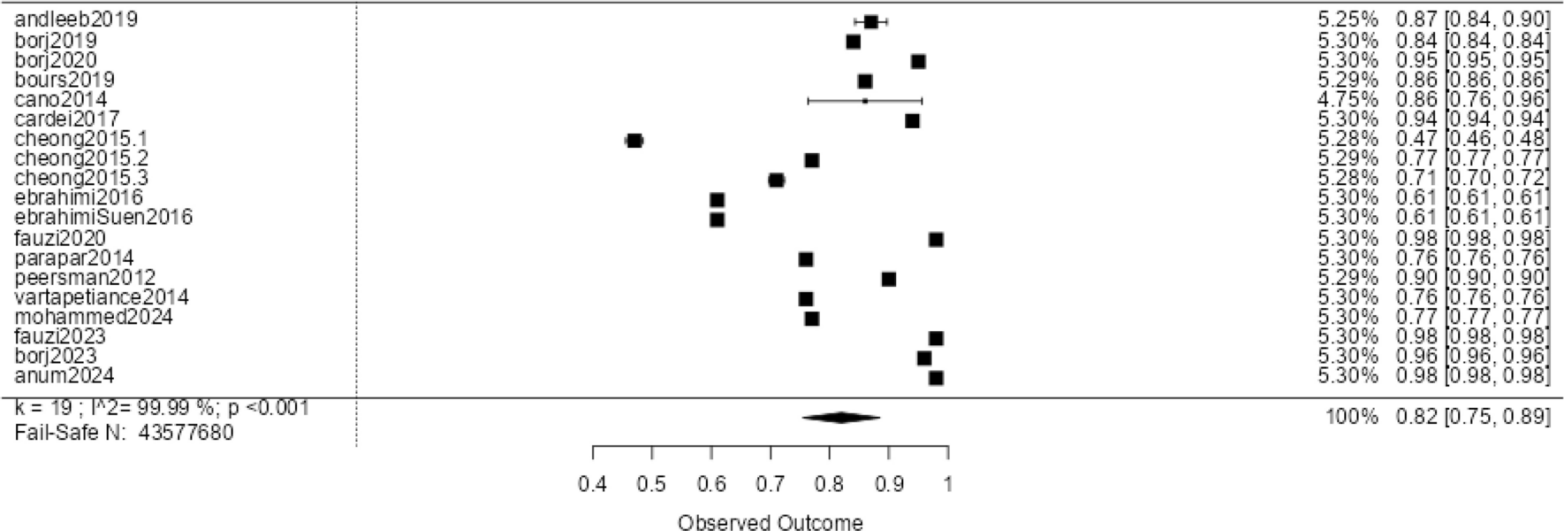
Forest plot for SVM using metric $\hbox {F}_1$.

Figure 6 shows a forest plot for the Support Vector Machine (SVM) algorithm using the F1 metric, revealing considerable variability across studies. F1 scores range from 0.98^54^ to 0.47^55^, with a meta-analytic average of 0.79 and a confidence interval of 0.72 to 0.87. Despite this variability, the plot indicates that SVM is generally robust in predicting the F1 metric.

Table 7 summarizes meta-analyses across various ML models and their performance metrics. The table highlights that the highest accuracy (0.92) is achieved by Multilayer Perceptron (MLP), while Random Forest (RF) excels in precision (0.99). However, RF’s lower recall and F1 scores suggest an imbalance in its predictive capabilities. In contrast, SVM shows a more balanced performance, with the highest F1 score (0.79), indicating better harmony between precision and recall.

**Table 7:** Comparative performance metrics of ML algorithms in grooming detection: summary of meta-analysis results.

Algorithm	ACC	P	R	F1
AB	0.62			
BiLSTM	0.95			
CNN			0.54	0.70
DT	0.89	0.75	0.56	0.62
KNN	0.81	0.68	0.64	0.45
LogR	0.83	0.76	**0.80**	0.72
MLP	**0.92**	0.81	0.78	0.79
NB	0.82	0.73	0.70	0.65
RF	0.81	**0.99**	0.25	0.30
SVM	0.88	0.86	0.72	**0.79**
GB	0.94			

Significant values are in bold.

## Discussion

What is the best ML algorithm for detecting grooming? In this critical field, the Support Vector Machine (SVM) emerges as an exceptionally effective algorithm. Central to its success is SVM’s ability to distinctly separate data classes, a vital feature when differentiating between harmless and potentially harmful communications. This separation is achieved through maximizing the margin around a defining hyperplane, greatly minimizing the risk of overfitting, a common challenge in analyzing complex, text-based data. SVM’s versatility is further showcased through its kernel trick, allowing it to deftly navigate the intricate, non-linear patterns often found in conversational data. This adaptability is crucial in handling high-dimensional spaces typical of text analysis in online chats, where the nuances of language require sophisticated interpretation. In the specific context of grooming detection, SVM’s balanced precision and recall capabilities, leading to a high F1 score, demonstrate its robustness and suitability for this sensitive and nuanced task, making it a preferred choice for safeguarding online interactions.

Delving deeper into the metrics associated with SVM in grooming detection, it is possible to discern key insights into its capability to accurately identify grooming perpetrators, and the implications of FPs and FNs. Firstly, the high precision (P) of SVM indicates a strong likelihood that its predictions of grooming incidents are correct. This is crucial for minimizing FPs, i.e., erroneously marking benign interactions as grooming. FPs are particularly problematic in this context, as they can lead to unwarranted accusations and undermine trust in the system.

The recall (R) metric of SVM, while not the highest among the algorithms, is still significant. Recall measures the model’s ability to correctly identify all actual grooming instances. A lower recall suggests that some genuine cases of grooming might go undetected (FNs). In the context of grooming, FNs are critical, as they mean potential perpetrators might not be identified, thus posing a risk to minors’ safety.

Most importantly, the F1 score of SVM, which is a harmonic mean of precision and recall, is the highest among the algorithms studied. This suggests that SVM achieves an effective balance in minimizing both FPs and FNs. In grooming detection, where accurate perpetrator identification and minimizing wrongful accusations are equally crucial, a high F1 score indicates optimal overall performance.

The metrics associated with SVM in Table 6 reflect its ability to detect grooming cases effectively and in a balanced manner. This underscores the importance of selecting a model that is not only precise but also maintains a balance between reducing FP and FN risks. These are critical elements for the successful implementation of online safety solutions, especially in sensitive areas like grooming detection, where the stakes are high both in terms of child safety and maintaining the integrity of the system.

In the context of grooming detection, both Multilayer Perceptron (MLP) and Support Vector Machine (SVM) have emerged as effective models, each with distinct strengths that suit specific challenges inherent to this sensitive problem.

MLP excels in recognizing complex, nonlinear patterns in text data, which is crucial for capturing the nuanced dynamics of online conversations often seen in grooming cases. Its architecture, with multiple layers of interconnected neurons, enables it to generalize well by capturing subtle feature interactions. Empirically, MLP has demonstrated high accuracy, reaching up to 92%, making it a powerful tool for this application. However, MLP’s “black box” nature and the computational cost associated with extensive parameter tuning present challenges, particularly in real-world scenarios where interpretability and efficiency are critical.

SVM is a robust model for classification tasks, especially effective in handling nonlinear data through the use of kernel functions. By optimizing the separation margin between classes, SVM can accurately distinguish between grooming and non-grooming interactions, achieving a recall of 72%, which is vital for minimizing false negatives in this context. SVM also benefits from faster prediction times and a solid mathematical foundation, making it reliable for real-time applications. Nevertheless, SVM requires careful selection of parameters, such as kernel type and regularization settings, which can also be computationally intensive.

In conclusion, MLP and SVM both offer unique advantages in grooming detection. MLP is ideal for capturing intricate patterns in rich, nonlinear data, while SVM provides a balanced approach with strong generalization and efficient computation. The choice between the two should be guided by the specific data characteristics and the application requirements, including the need for interpretability and computational resources. The No Free Lunch Theorem further underscores that neither model is universally superior, emphasizing the importance of tailored model selection for optimal performance.

### Limitations and future directions

This study focuses specifically on the application of Machine Learning (ML) techniques for detecting online grooming through text-based interactions. One limitation of our research is the exclusion of physical cues, such as gesture detection, eye-gazing, or other non-verbal signals commonly associated with harassment and abuse. The primary reason for this omission is that our work is centered on online grooming, which predominantly relies on linguistic and conversational strategies rather than physical behavior. Detecting physical cues would require a different methodological approach involving video or image analysis, which lies outside the scope of this study. By narrowing our focus to textual analysis, we aimed to address the pressing need for effective online grooming detection tools, given the increasing reliance on digital communication among children and the unique challenges posed by text-based manipulation tactics.

It is worth highlighting that only a limited number of studies have utilized datasets other than PAN12 or Perverted Justice (e.g.,^44,55,56,81,82^). Additionally, the PAN12 dataset is based on conversations that date back to before 2012, which may limit its relevance to current grooming tactics. Even when widely used datasets like PAN12 and Perverted Justice were cited, they were often augmented with additional data or only subsets were used, adding another layer of complexity to the interpretation of results. This variability in data handling emphasizes the challenges in ensuring consistency and comparability across studies in this research domain.

In the interest of continual improvement in cybersecurity, we recommend updating existing databases or making new ones available to the scientific community to further enhance the prediction and detection of grooming cases.

Achieving this would have two main consequences. First, it would provide more studies from diverse databases, allowing for additional complementary analyses, such as sensitivity analysis. Sensitivity analysis would be particularly relevant here, as it would enable us to assess the stability and robustness of results with the inclusion of new samples. This analysis becomes especially valuable as the number of studies increases, allowing for a more comprehensive examination of variability across studies and strengthening model precision by accounting for a broader range of contexts.

On the other hand it’s also essential to be aware that the databases analyzed here represent only the online dimension of grooming. Grooming is a more complex process that encompasses other stages not necessarily occurring online. However, having this information can prevent grooming cases, given the current significance of online media among children. Nevertheless, caution is advised when comparing grooming strategies between available online data and data from victims offline^4^. Given the evolving nature of online behaviors and digital platforms, future datasets should aim to capture more contemporary and diverse patterns of grooming. This would enable the development of more robust algorithms, enhancing the relevance and accuracy of detection models in real-world scenarios.

In this way, future research should integrate a nuanced understanding of the trauma-related impacts of grooming and online sexual exploitation, drawing on systematic review and meta-analytic findings that underscore the need for robust, context-sensitive models. Similar to findings in another systematic review, though not meta-analytic, supervised learning approaches such as SVM remain predominant in detecting grooming behavior. Recent studies have begun implementing deep learning methods, particularly in contexts of online harassment and abusive behavior. However, deep learning applications remain limited due to the scarcity of large, representative datasets, highlighting the critical need for diverse and realistic user data to enhance algorithmic validation and practical applicability^83^.

Applying a psychosocial impact of trauma framework^84^ to grooming detection could help address these limitations. Grooming, as a form of sexual abuse, creates distinct trauma for victims in either physical or virtual settings. This trauma is context-specific and mediated by factors related to both perpetrator and victim factors. First, grooming offenders are often male^85^ and may be acquaintances, family members, or even peers of the victim, not exclusively adults^86^. These offenders commonly assess victim vulnerability through probing questions, learning about family routines, caregiver availability, and potential signs of conflict or lack of supervision. Such assessments allow offenders to adapt their tactics, aligning with the victim’s emotional needs to increase engagement opportunities while minimizing external interference.

Strategically, perpetrators leverage these details to build exclusivity and trust. Male perpetrators may employ gifts, praise, or promises to present themselves as unique and irreplaceable, while female perpetrators often adopt caregiver or mentor roles, fostering a misleading sense of support^85^. Although women represent only about 2% of reported sexual abuse cases, this percentage may be underestimated. The subtlety of these tactics and the tendency to underreport abuse committed by female offenders often allow such incidents to go unnoticed within trusted contexts. Each relational manipulation provides the perpetrator with increasing control over the victim, discouraging disclosure. Once trust is established, sexual exploitation begins, often progressing from sexually suggestive language to explicit physical or virtual contact. Importantly, online environments can accelerate these strategies due to their anonymity and limited nonverbal cues^87^, where offenders can simultaneously contact multiple children and select vulnerable targets, leveraging threats only sparingly to maintain compliance.

The setting in which child sexual abuse occurs involves critical elements worth examining. Offenders who operate exclusively in virtual contexts (e.g., through child pornography)^88^ are generally less likely to engage in physical contact with minors. This is likely due to lower levels of antisocial traits, greater empathy for victims, and stronger psychological barriers that inhibit the transition from fantasy to physical action. They also tend to have fewer prior sexual offenses, lower incidences of mental health issues, substance abuse, and social skill difficulties, factors that may serve as protective elements. These offenders are often younger, more educated, and have higher incomes than contact offenders, and they are more likely to lack cohabiting relationships. However, they exhibit significant pedophilic interests and may face fewer challenges in regulating sexual behavior but are often limited in physical access to minors. In contrast, offenders who engage in both virtual and physical abuse combine high internet access with pronounced antisocial traits.

Finally, the vulnerability of potential victims, particularly children from marginalized backgrounds, requires consideration. Young girls, individuals from minority groups, and those with histories of sexual or domestic violence, post-traumatic stress, or emotional dysregulation are particularly vulnerable to child sexual exploitation^89^. Additional risk factors include hopelessness, suicidality, depression, sexually explicit behavior, rule-breaking tendencies, substance use, school challenges, and unstable family environments. Recognizing these indicators is essential not only for protective measures against grooming but also for processing the trauma should abuse occur. This meta-analysis, with its focus on ML in detecting grooming, underlines the necessity of contextualized, trauma-informed models, calibrated to real-world user data to effectively mitigate these risks.

## Methods

This systematic review was carried out in accordance with the methodology outlined by the Preferred Reporting Items for Systematic Review and Meta-Analysis (PRISMA) guidelines^90,91^. For example, this guideline has been applied in a scoping review on the use of vision transformers for skin cancer detection and diagnosis^92^.

### Inclusion criteria

The criteria for excluding or including articles are as follows: 1) The study must conform to the definition of grooming, and 2) The article must consider machine learning algorithms for grooming detection. Studies that implement other types of algorithms (e.g., mathematical models) will be excluded from the review. These two criteria are not mutually exclusive; both must be met for an article to be included in the review.

#### Search strategy

The articles were obtained from five databases: Web of Science (WoS), Scopus, IEEE (Institute of Electrical and Electronics Engineers), Springer, and PubMed, by searching the entire document without year filters. The same search strategy was used across all databases, utilizing the following keywords: Predatory conversation, Grooming recognition, Child grooming, Grooming Detection, Sexual predation, and Online grooming. For example, the search in WoS was conducted as follows: ALL=(“Predatory conversation”) OR ALL=(“Grooming recognition”) OR ALL=(“Child grooming”) OR ALL=(“Grooming Detection”) OR ALL=(“Sexual predation”) OR ALL=(“Online grooming”). The details of the search strategy according to the PICO method (Participants, Intervention, Comparator, and Outcomes) are provided in Table 8^93^.

**Table 8:** Search strategy according to the PICO mthod.

Aspect	Description
Participants	Includes studies focused on victims child grooming in online and offline contexts, particularly where machine learning (ML) is used for the detection of predatory conversations.
Intervention	Evaluation of ML models applied to detect grooming and predatory behaviors on digital platforms, using algorithms (e.g., SVM, CNN) and others for the classification of suspicious conversations.
Comparator	No direct comparator was used, but the studies included in the review were compared post hoc in terms of their results across various metrics (ACC, P, R, F1) to analyze the effectiveness of each model.
Outcomes	Effectiveness of ML models for detecting grooming, measured through metrics such as accuracy, F1-score, recall, specificity, and others derived from the confusion matrix.
Limitations	No year filters were applied, which might affect the studies’ temporal relevance, though most are recent due to the topic. No restrictions on language or region were imposed, but some databases may have limited study availability.

#### Data extraction and studies selection

Also, it is imperative to ascertain the number of non-significant studies capable of potentially influencing the overall results of the meta-analysis. This determination is achieved by calculating the Fail-Safe N (FSN), which takes into account the quantity of studies included in the meta-analysis, multiplied by a factor of 5, and then augmented by 10. If the calculated FSN value surpasses this threshold, it signifies that the number of studies included is sufficient to ensure the robustness of the meta-analysis findings. These essential considerations contribute to the precision and validity of the meta-analysis process^94^. Certainly, including a minimum of two studies is a prerequisite for a meta-analysis. It’s worth noting that in all 10 meta-analyses conducted here, the calculated Fail-Safe N (FDN) surpasses the threshold, ensuring an ample number of studies.

#### Quality assessment

For this report, the quality review consists of 8 questions inspired by the Critical Appraisal Skills Programme (Critical Appraisal Skills Programme. Casp checklists. https://casp-uk.net/casp-tools-checklists/, 2023). The questions are assessed using a binary scale. Below is a list of the questions used: QA1 Does the document clearly specify the context of the research on the topic of grooming? QA2 Is the machine learning algorithm used in the research clearly specified? QA3 Is the dataset used in the study appropriately described? QA4 Is the data analysis process detailed, including data acquisition, exploration, transformation, modeling, and validation? QA5 Is the validation method adequate? QA6 Are the metrics appropriate for the grooming problem being addressed? QA7 Is the finding of the study specified? QA8 Are the limitations of the study specified?. Articles classified as ’High’ (7-8) stand out for their completeness and methodological rigor. Those classified as ’Medium’ (4-6) present opportunities for improvement in the clarity and detail of their presentation. Articles described as ’Low’ (0-3) meet few or none of the evaluation criteria and require considerable revision to reach the necessary standards of clarity and detail.

#### Statistical analysis

To conduct the meta-analysis, the R software was used along with the Metafor package, which is based on effect size (ES). Since some studies do not report variance directly, variances were calculated using appropriate metrics such as accuracy, precision, recall, F1-score, and specificity, as well as the number of data points used to evaluate the model. Equation (6), for instance, computes the variance of a binomial distribution, where *p* signifies the probability of success in the specific case under study (Grooming).

6 $$\begin{aligned} \textsf {v}_{\textsf {p}}=\frac{\textsf {p}*(1-\textsf {p})}{\textsf {N}} \end{aligned}$$

This function allows for the integration of results from multiple studies to obtain a combined effect size and its associated variance. Thanks to the Effect Size (ES) and its variance, each of the 33 meta-analyses reported here was possible. In this context, each meta-analysis is accompanied by a graphical representation known as a ”forest plot.” The final result of the meta-analysis is shown, visualizing various pieces of information, including the total number of experiments (k), heterogeneity ($\text {I}^{2}$), p-value, and Fail-Safe N (FSN). The meta-analysis results were interpreted considering the heterogeneity between studies, evaluated using the $\text {I}^{2}$ statistic, and the statistical significance of the combined effect sizes.

## Data Availability

The datasets created and/or examined in this study are accessible from the corresponding author upon reasonable request.
